# Early Outcomes of Roux-en-Y Gastric Bypass in a Publically Funded Obesity Program

**DOI:** 10.1155/2013/296597

**Published:** 2013-08-21

**Authors:** Kevin A. Whitlock, Richdeep S. Gill, Talal Ali, Xinzhe Shi, Daniel W. Birch, Shahzeer Karmali

**Affiliations:** ^1^Faculty of Medicine & Dentistry, University of Alberta, Edmonton, AB, Canada T6G 2R7; ^2^Department of Surgery, University of Alberta, Edmonton, AB, Canada T6G 2B7; ^3^Center for the Advancement of Minimally Invasive Surgery (CAMIS), Royal Alexandia Hospital, Edmonton, AB, Canada T5H 3V9

## Abstract

* Background*. There is limited literature assessing the outcomes of bariatric surgery in a publically funded, North American, multidisciplinary bariatric program. Our objective was to assess outcomes of roux-en-Y gastric bypass (RYGB) in a publically funded bariatric program through a retrospective review of patient records. *Methods*. 293 patients spent a median of 13 months attending a multidisciplinary obesity clinic prior to undergoing laparoscopic RYGB surgery. The hospital was a Canadian, publically funded, level 2 trauma center with university teaching services. *Results*. 79% of the patients were female and the average BMI at first visit to clinic was 55.3 kg/m2. The average decrease in BMI was 19.2 ± 0.9 kg/m^2^. This was an average absolute weight loss of 56.1 kg or 35.5% of initial weight. The average excess weight loss was 63.4 ± 20.4%. Improvement or resolution of obesity related comorbidities occurred in 65.9% of type 2 diabetics and in 50% of hypertensive patients. *Conclusion*. Despite this being an unconventional setting of a publically funded program in a large Canadian teaching hospital, early outcomes following RYGB were appropriate in severely obese patients. Ongoing work will identify areas of improvement for enhanced efficiencies within this system.

## 1. Introduction

Globally, the body mass index (BMI) has been increasing over the last 30 years [1]. Currently, more than 20% of the world population is overweight and approximately 10% are obese [2]. In particular, the World Health Organization estimates that over 1.5 billion individuals are overweight and 500 million are obese [3]. In Canada, an estimated 60% of the population was classified as overweight and 24% as clinically obese [4–7]. Currently, bariatric surgery is the only evidence-based approach shown to be effective at achieving marked and sustained weight loss in obese individuals [8]. In addition, resolution of comorbidities, such as type 2 diabetes mellitus (T2DM) and hypertension commonly, occurs following bariatric surgery [9].

Roux-en-Y gastric bypass (RYGB) is the most commonly performed bariatric surgical procedure in North America. Weight loss was thought to occur by malabsorption; however, this may not be the primary mechanism of action. Recently, there has been a suggestion that rapid nutrient delivery to the jejunum and ileum may trigger a hormonal response (involving GLP1 and PYY) that alters satiety and hunger [10, 11]. A number of studies have shown significant and sustained weight loss following RYGB in severely obese individuals [8]. However, to date, there have only been a small number of studies that have looked at RYGB outcomes in a publically funded Canadian healthcare system [12–15]. The objective of this study was to analyze the outcomes of laparoscopic RYGB performed as part of a comprehensive weight management program in a publically funded Canadian hospital.

## 2. Methods

### 2.1. Study Design

This was a retrospective observational study to investigate the outcomes following RYGB as part of a comprehensive weight management strategy. Weight Wise is a multidisciplinary adult weight management program, which includes physicians, nurses, dieticians, and other healthcare professionals. Obese patients are enrolled and followed in the Weight Wise clinic, with bariatric surgery offered to patients as part of an overall treatment strategy by the multidisciplinary team. Ethics approval was obtained from Human Research Ethics Board at the University of Alberta. All data was collected from health records and reviewed within a 3-month time frame. A total of 293 patients who underwent laparoscopic RYGB since the inception of a bariatric program were reviewed from Jan 2005 to June 2011.

The hospital was a Canadian, publically funded, level 2 trauma center with 750 inpatient beds. The center was located in the inner city and had university-based teaching services, including an accredited minimally invasive surgery/bariatric program. Three generalist surgeons performed all the operations with the MIS fellow present at each surgery since 2010.

### 2.2. Data Recorded

Data collection was performed by two individuals. Demographic information including preoperative weight, height, and age was recorded. The presence of preoperative medical conditions was also recorded, including T2DM, hypertension, sleep apnea, and gastroesophageal reflux disease (GERD). Operative details were extracted including operative time, position of roux limb, use of OrVil stapler (Covidien; Massachusetts, USA), perioperative complications, and use of hemostatic agents. Patient records at the Weight Wise medical program were used to extract postoperative outcomes, including change in weight, complications, reoperations, change in medications, resolution of medical conditions, and diagnostic investigations. The postoperative time points were 1 month, 3 months, 6 months, 1 year, 2 years, and 3 years.

### 2.3. Outcomes

The data was analyzed to determine changes in absolute weight, body mass index, and excess body weight. Weight values at the initial entry into the Weight Wise program were compared to preoperative and postoperative weights.

Resolution or improvement of obesity-related comorbidities following RYGB was recorded. For T2DM, resolution was defined as cessation of medications and normalization of blood glucose values. Improvement in T2DM was defined as any decrease in the amount or number of oral hypoglycemic agents or insulin compared to the preoperative period. For hypertension, resolution was defined as a cessation of medications and normalization of blood pressure. Improvement was defined as any decrease in the amount or number of antihypertensives compared to the preoperative period. Resolution of GERD was defined as cessation of medications and improvement in symptoms. Development of GERD was defined as the initiation of proton pump inhibitors postoperatively. Median time to resolution was calculated based on the postoperative time of visit at which a change in comorbidity status was first recorded in the clinic.

The mean operative time was compared among RYGB performed near the inception of the program and those performed recently. The perioperative complication rate was calculated for gastrointestinal hemorrhages, anastomotic leaks, stricture formation, ulceration, reoperation, and mortality. Perioperative gastrojejunal (GJ) leaks were diagnosed with an intraoperative methylene blue leak test. Patients with symptoms of gastric outlet obstruction were evaluated with a gastrografin upper GI series followed by esophagoduodenoscopy for definitive diagnosis and treatment. Length of hospital stay was calculated from the date of surgery to the date of discharge.

The rates of complications associated with retrocolic and antecolic roux limb position were determined. The use of an OrVil stapler was compared to suturing for the GJ anastomosis with respect to postoperative stricture development. 

Cost analysis was performed with a sample year of 2010 and values were adjusted by 5% for inflation per year. Weight Wise clinic costs are an average value for either a preoperative or postoperative visit. The average cost of a RYGB surgery is based on the supply cost per case and labour cost for average OR time. Length of stay cost is based on the total average inpatient cost per day at the Royal Alexandra Hospital. The overall cost analysis is based on the average uncomplicated patient who required no additional investigation due to postoperative complications or reoperations.

### 2.4. Treatment Details

All patients were enrolled in an adult weight management program called Weight Wise. The program consisted of a multidisciplinary team of physicians, nurses, dieticians, and counselors who were experienced in the treatment of obesity. The clinic assisted with diet and lifestyle modifications and provided follow-up care after the surgery. Weight management plans involved diet and behavior modification, counseling, education, advice on exercise, and pharmacologic treatment. When patients showed progress in the clinic, they were presented with the option of surgery. Patients were presented with multiple surgical options according to their unique situation; RYGB was only one of the options. Following the Canadian guidelines, patients older than 18 years of age with a BMI > 40 kg/m^2^ or a BMI > 35 kg/m^2^ with comorbidities were selected for surgery. Although in practice, due to limited resources and a large population that the clinic serves, patients with a higher BMI are prioritized, leading to increased wait times for others. All patients are nonpaying in the publically funded system, and thus resource allocation remains an important issue.

After surgery, the patients were followed up with the program at specific intervals to monitor their postoperative progress. When patients achieved adequate weight loss without complications, they were transitioned from the clinic to the care of their family doctor. Patients returned to the clinic if they had subsequent complications or other failures of treatment relating to their weight. Most often patients are transitioned 2 years after surgery.

### 2.5. Surgical Technique

All RYGB procedures were laparoscopic. Conversion to open surgery occurred if the procedure could not be completed laparoscopically. The gastrojejunal (GJ) anastomosis was created either by intracorporeal suturing (hand-sewn) or the use of circular EEA (OrVil) stapler. The enteroenterostomy was created with staplers and suturing. The roux limb was approximately 100 cm in length and was placed either antecolic or retrocolic depending on the judgment of the surgeon. Methylene blue injected through a gastroscope or nasogastric tube was used to test the integrity of the GJ anastomosis.

### 2.6. Statistical Analysis

Body mass index was calculated based on the absolute weight (kg). Excess weight was calculated as the amount of weight over a BMI of 24.9 kg/m^2^ preoperatively. Excess weight loss (EWL) was determined by the weight loss at a specific postoperative time point. Wilcoxon Signed Rank test and paired *t*-test were used for continuous variables according to data normality. Fisher exact test was used for proportion differences.

## 3. Results

Patient demographics are shown in Table 1. Initial weight and BMI were recorded at first presentation to the bariatric clinic. The median number of visits to the Weight Wise program prior to surgery was 9, over a median period of 13 months. Preoperative weight and BMI were recorded just prior to the RYGB surgery. The preoperative BMI was significantly lower than the initial BMI (51.6 ± 9.4 versus 55.3 ± 10.0 kg/m^2^, *P*-value <0.001). The median follow-up time was 1 year postoperatively; 94.5% of the patients were followed up with the Weight Wise program at some point postoperatively. At 1 year, 69.3% of the patients were followed up with the bariatric program. At 3 years follow-up, 22.2% of the patients continued with the Weight Wise program. However, patients normally transition to general practitioner care around 2 years postoperatively if they experienced consistent weight loss and minimal complications.

Operative data is summarized in Table 2. There was no significant difference in operative times among the first hundred and the second hundred cases or the last 93 cases. The majority of RYGB surgeries were teaching cases, and since 2010, an MIS fellow has been present and performing a portion of the operation. Furthermore, the addition of a third generalist surgeon and the change to the OrVil stapler for GJ anastomosis (which occurred at the same time) did not significantly affect operative times. 

Intraoperative complications occurred at a rate of 4.8%. The seven patients (2.4%) had a GJ leak, 4 patients (1.4%) had a stomach stapler line bleed, and 3 patients (1.0%) had abdominal bleeds. There were no intraoperative deaths.

Surgeon preference determined whether the antecolic or retrocolic positioning of the roux limb was chosen, with an increased frequency of retrocolic positioning (60%). A percentage of 5.3 of the patients had gastrointestinal obstruction after retrocolic positioning versus 7.9% of the patients who had antecolic roux limb positioning. This difference did not reach statistical significance. 

Table 3 summarizes the postoperative complications with respect to the type of GJ anastomosis. Both the 21 mm and 25 mm OrVil staplers were used in the study, but the stapler group does not differentiate between the two. The sewn group had significantly more GJ leaks when compared to the stapler group (8.8% versus 0%, *P*-value <0.001). There were no differences in the rates of GJ stricture, abdominal bleeds, staple-line bleeds, or upper GI obstruction when comparing the two groups.

**Figure 1:**
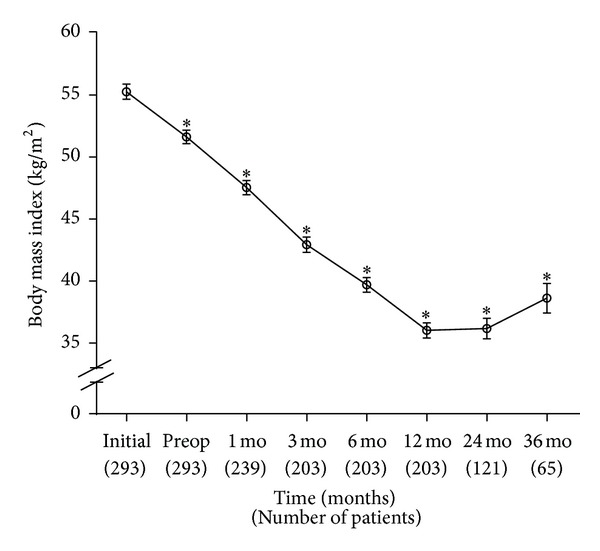
Average body mass index (BMI) from initial visit (first preoperative visit to bariatric clinic) to 36 months (mo) postoperatively. RYGB surgery occurred just after preoperative (Preop) BMI. Preop BMI was compared to initial BMI. All postoperative BMI values were compared to the Preop BMI value. No statistically significant increase from 24 to 36 months postoperatively. *P value <0.001. Number of patients at each time point is shown below the *x*-axis.

As shown in Figure 1, BMI significantly decreased from the initial visit at the Weight Wise to the preoperative assessment with only lifestyle modifications. There was a significant decrease in the mean BMI at 1 month after RYGB compared to preoperative assessment (*P*-value <0.001). Furthermore, there was a statistically significant decrease in BMI at each postoperative time point when compared to the preoperative value (*P*-value <0.001). The mean decrease in the BMI was 19.2 ± 0.9 kg/m^2^ from the initial visit to 12 months after surgery. There was no significant increase in BMI between years 2 and 3 postoperatively. The average decrease in BMI was 16.7 ± 1.4 kg/m^2^ for the patients who were followed up for 3 years postoperatively.

**Figure 2:**
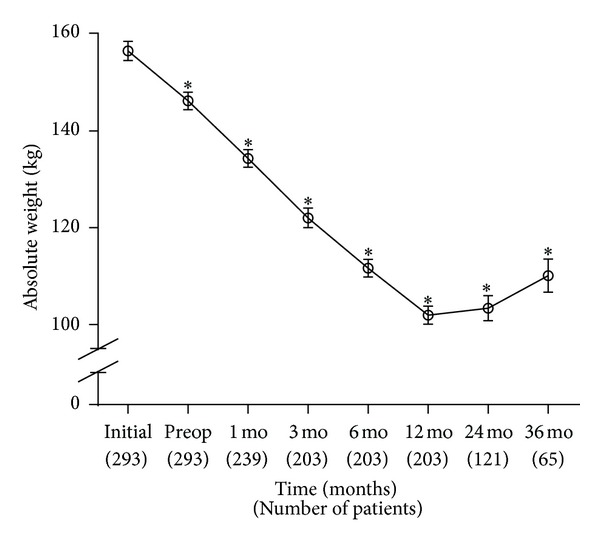
Average absolute weight (in kg) from initial visit to 36 months (mo) postoperatively. RYGB surgery occurred just after preoperative (Preop) weight. Preop weight was compared to initial weight. All postoperative weight values were compared to the Preop weight value. No statistically significant increase from 24 to 36 months postoperatively. *P value <0.001. Number of patients at each time point is shown below the *x*-axis.

As shown in Figure 2, the absolute weight (kg) showed significant decreases at all postoperative time points when compared to the preoperative value (*P*-value <0.001). This was comparable to the change in BMI seen in Figure 1. On average, patients lost 56.1 ± 17.7 kg or 35.5 ± 8.6% of the initial weight at 12 months after RYGB. This was equivalent to an excess weight loss of 63.4 ± 20.4% at 12 months after RYGB. As with BMI, there was no significant increase in the weight from 2 years to 3 years postoperatively.

Postoperative complications are shown in Table 4. There were two deaths (0.7%) postoperatively that were related to ongoing sepsis, 15.4% of the patients experienced a complication within the first 3 months postoperatively, 4.8% of the patients experienced a GJ leak, and 6.5% of the patients experienced a GJ stricture after laparoscopic RYGB.

A percentage of 4.7 of the patients required surgical revision of the RYGB. These included bowel resection due to obstruction (1.7%), gastrogastric fistula repair (1.4%), GJ anastomosis revision secondary to anastomotic leak (1.0%), enteroenterostomy repair (0.3%), and stomach repartitioning (0.3%). Fifty-eight patients (19.8%) underwent a subsequent panniculectomy at a medium of 24 months after RYGB to remove excess skin after their significant weight loss.

Table 5 summarizes the prevalence of obesity-related comorbidities before and after RYGB surgery. Improvement, resolution, and development of these complications were previously defined (see Section 2). Cost analysis is summarized in Table 6. The average cost for a patient to go through the preoperative visits, RYGB surgery, and the postoperative visits is $24,742.88.

## 4. Discussion

Our study demonstrates that RYGB can be performed safely and effectively on super obese individuals in a publically funded Canadian academic center as part of a comprehensive weight management strategy. Super obese patients at our institution had marked and sustained weight loss at three years post-RYGB, with acceptable morbidity. 

The Weight Wise obesity program is unique in that it services a very large region due to limited centers in its proximity. Patients are nonpaying, and those selected for surgery tend to have a higher BMI. Patients with higher BMIs are preferentially selected because they are deemed more likely to benefit from bariatric surgery. This biases the surgical candidates towards those with more significant comorbidities and likely impacts the results we obtain. Additionally, nearly 25% of our patients are unemployed, on social assistance, or on long term disability [16]. This is in contrast to private centers where these same patients would not be able to afford multidisciplinary treatment and RYGB surgery. However, even with a unique patient population, this study shows that a multidisciplinary program in a public system can still obtain good results. 

Compared to privately funded centers, our patients had a higher preoperative BMI. The mean BMI just prior to surgery in our study was 50 kg/m^2^, which is demonstrative of the bias mentioned previously. In fact, one-fifth of the patients in this study had a BMI greater than 60 kg/m^2^. In contrast, Adams et al. reported a mean preoperative BMI of 44.9 ± 7.6 kg/m^2^ in their study of a privately funded bariatric practice [17]. Regardless of this difference, we were still able to obtain significant and comparable weight loss.

The mean weight loss and decrease in BMI following RYGB in our series were comparable to the literature [12, 18, 19]. Kothari et al. recently reported data from a similar institution in the private American sector [20]. Over a seven-year period, their academic center performed 700 RYGB surgeries and reported excess weight loss of 72.4% at 12 months postoperatively, which was comparable to our 63.4%. These authors also reported a weight gain trend in the latter years, nearly 60 months of follow-up [20]. This was similar in our study, in which a plateau was observed at approximately 12 months postoperatively. Additionally, Welch et al. reported a mean excess weight loss of 59.1% and a decrease in BMI of 15.7 kg/m^2^, which was comparable to our 16.7 kg/m^2^ decrease after 36 months [21]. Therefore, in comparison to American academic centers, our study demonstrates that similar effectiveness of RYGB can be achieved in a publically funded Canadian health care system.

The mean operating time to perform laparoscopic RYGB in our series was similar to that previously reported in the literature [13, 19, 20, 22, 23]; however, considerable variability exists between studies [18, 24, 25]. Furthermore, a number of studies have suggested that a learning curve exists and influences operating times [13, 25, 26]. The Canadian study by Schaeffer et al. reported that their operative time was significantly lower in their second cohort of 60 patients, with a mean time of 146 min [13]. However, our series did not demonstrate a discernible learning curve. This may be related to the frequent turnover of residents and fellows commonly participating in the operating room at an academic center. In terms of the bariatric surgeons at our institution, they all have received fellowship training, which may influence operative times. Supportively, Oliak et al. suggest that fellowship training in laparoscopic RYGB surgery appears to decrease operating times [24]. 

Overall, our complication rates following RYGB were comparable to other laparoscopic RYGB series [13, 25, 26]. However, we observed an overall GJ stricture rate of 6.5%, compared to a 2–4% rate reported in the literature [27–32]. Interestingly, the rate of GJ stricture was lower at 4.7% in the patients whose GJ anastomosis was secured with a circular stapler. This is comparable to the recent American study by Dolce et al. who reported a GJ stricture rate of 9.4% after using a circular stapler for the GJ anastomosis [33]. Furthermore, our rate was comparable to the Swiss study by Suter et al. who reported a GJ stricture rate of 5.8% after 466 RYGB surgeries [34].

The most important complication following RYGB remains to be the anastomotic leakage, specifically the GJ anastomosis. The American study by Fernandez Jr. et al. identified 580 patients who underwent RYGB surgery at a privately funded institution [35]. They reported a comparable postoperative GJ leak rate of 4.1%. Moreover, their mortality rate was 0.7% for the laparoscopic RYGB group, which was comparable to our mortality rate. Therefore, our study has comparable serious complication and mortality rates to a privately funded institution.

Our series reported an overall improvement in T2DM of 65.9%. This is comparatively low in relation to the large American study by Schauer et al., where they found that 80% of their 191 diabetic patients showed improvement after laparoscopic RYGB [36]. Improvement in their study was defined as a decrease in oral antidiabetic agents, which is similar to our definition. In contrast, the Canadian study by Schaeffer et al. reported a 16% decrease in the rate of T2DM after RYGB [13]. This was equivalent to about a 50% improvement rate. These differences in T2DM improvement rates are likely multifactorial. 

Interestingly, the rate of improvement of hypertension in our study was 50%, which was higher than that reported by Batsis et al. at a privately funded institution. Batsis et al. reported that approximately 40% of their hypertensive patients improved after RYGB surgery [37]. The Canadian study by Schaeffer et al. also reported a 19% decrease in the rate of hypertension after RYGB, which was approximately a 40% improvement rate [13]. So, there is discrepancy in the improvement rates for both T2DM and hypertension. The variations in reported rates in the literature likely relate to variation of the definition of improvement, and how intensively these improvements are searched for. For instance, our study did not systematically assess comorbidities at each follow-up visit, which may have led to underestimation of the actual improvement rates. Nonetheless, it is widely accepted that RYGB surgery can improve obesity-related comorbidities [9]. 

Cost analysis is a very important aspect of surgeries performed in a publically funded health care system. A recent study by Martin et al. reported that private clinics in Canada were charging an average of $16,000.00 to patients for a RYGB procedure [38]. Compared to our average cost this is significantly less. However, it is important to consider that their number of preoperative visits (2.7) and postoperative visits (3.7) was less than our clinic. This translates into fewer visits with the multidisciplinary team (including dieticians, psychologists, and education classes). Consequently, patients receive more comprehensive care through our clinic compared to private Canadian clinics. Interestingly, our total cost is comparable to many American centers. Salem et al. performed a cost analysis of both RYGB and adjustable gastric banding surgeries [39]. They obtained data from national hospital databases representing a private patient payer model. The centers they included were large hospitals compared to ours. Their reported cost ranged from $18,000 to $36,000 for one patient to undergo RYGB surgery and postoperative follow-up [39]. Additionally, they found that RYGB had a $14,680/QALY (Quality Adjusted Life Year). Based on the assumption that <$25,000/QALY defines cost effectiveness, then RYGB is a cost effective procedure. Their QALY analysis took into account the benefits of RYGB on the overall health and the resulting future cost savings. Consequently, at our current cost per patient we can offer a cost effective, publically funded RYGB surgery.

There are several limitations to consider. Firstly, the retrospective nature of our study may lead to selection bias. Secondly, at a 3-year follow-up after RYGB, only 23.5% of the patients remained. However, patients normally transition to their general practitioner's care if they experienced consistent weight loss and minimal complications. The transition usually occurs when their weight stabilizes at approximately 2 years after RYGB. If patients experienced complications, they would be referred back to the clinic. Thirdly, it is difficult to consider all the possible variables in a public system that may influence our patients and this study. Consequently, the overall effectiveness of the program can be influenced by variables not considered in this study. Lastly, our setting of a trauma hospital is unconventional for a bariatric program. The surgeons in the study do have fellowship training, but they have diverse practices within the trauma center as general surgeons in addition to performing bariatric surgery. However, even with the limitations identified, this study demonstrates that significant short and intermediate term weight loss is achieved for super obese patients following RYGB.

In conclusion, our study demonstrates that RYGB performed in a publically funded bariatric program, in the setting of a teaching hospital, whose surgeons have diverse practices outside of bariatric surgery, is still effective in producing significant and sustainable weight loss.

## Figures and Tables

**Table 1:** Patient demographics for laparoscopic RYGB.

*N* total	293
*N* female	231 (79%)
*N* male	62 (21%)
Age	41.6 ± 9.3 years
Initial weight	156.6 ± 32.2 kg
Initial BMI	55.3 ± 10.0 kg/m^2^
Preoperative weight	146.2 ± 30.6 kg
Preoperative BMI	51.6 ± 9.4 kg/m^2^

**Table 2:** Operative data for laparoscopic RYGB (*n* = 293).

OR time total	161.1 ± 45.6 min
OR time (1–100 cases)	158.8 ± 43.7 min
OR time (101–200 cases)	158.4 ± 41 min
OR time (201–293 cases)	166.5 ± 51.9 min
OR time (1–155 cases*)	161.2 ± 42.7 min
OR time (156–293 cases**)	161.1 ± 48.9 min
Converted to open RYGB	3 (1%)
Antecolic GJ anastomosis	117 (40%)
Retrocolic GJ anastomosis	172 (60%)
GJ sewn	161 (56%)
GJ stapler	128 (44%)
LOS (mean)	5.4 ± 10.9 days
LOS (median)	3 days

*Two surgeons and hand-sewn GJ.

**Three surgeons and change to OrVil stapler for GJ.

**Table 3:** Hand-sewn versus stapled GJ anastomosis in laparoscopic RYGB.

Patient *n* = 288	Hand-sewn GJ (*n* = 160)*	OrVil stapler GJ (*n* = 128)^†^	*P* value (Fisher exact test)
Stricture	13	6	0.243
Leak	14	0	0.001
Abdomen bleed	4	5	0.496
Staple-line bleed	1	1	0.874
Upper GI obstruction	10	3	0.113

*One robotic sewn, 4 missing data.

^†^Hand-sewn GJ anastomosis versus 21 mm and 25 mm OrVil stapled anastomosis.

**Table 4:** Postoperative complications after laparoscopic RYGB.

	Early (*n* < 3 months)	Late (*n* > 3 months)	Total *n* (%)
Mortality	2	0	2 (0.7)
Stomach staple-line bleed	2	0	2 (0.7)
Abdominal bleed	9	0	10 (3.7)
GJ leak	13	1	14 (4.8)
GJ stricture	11	8	19 (6.5)
Upper GI Obstruction	8	5	13 (4.4)
Overall (*n*, %)	45 (15.4)	14 (4.8)	59 (26.1)

**Table 5:** Obesity-related comorbidities before and after laparoscopic RYGB.

Comorbidity	Preoperative (*n*, %)*	Resolved (*n*, %)**	Improved (*n*, %)**	Developed (*n*, %)*	Median time to improvement (months)
T2DM	82 (28.0)	41 (50.0)	13 (15.9)	0 (0)	3
Hypertension	145 (49.5)	54 (37.0)	19 (13.0)	0 (0)	6
GERD	89 (30.4)	9 (10.1)	0 (0)	18 (6.1)	6

*Percentage of total patients.

**Percentage of patients with the respective comorbidity preoperatively.

**Table 6:** Cost analysis for laparoscopic RYGB surgery in a publically funded system.

Item	Cost per item ($)	Average number of items per patient	Total cost of item ($)
Preop visit	369.00	8.75	3,228.75
RYGB surgery	6,707.06	1	6,707.06
One day hospital stay	3,600.00	3.7	13,320.00
Postop visit	369.00	4.03	1,487.07

Total per patient			24,742.88
